# Improving Performance of Electrospun Nylon 6,6 Nanofiber Membrane for Produced Water Filtration via Solvent Vapor Treatment

**DOI:** 10.3390/polym11122117

**Published:** 2019-12-17

**Authors:** Nur Syakinah Abd Halim, Mohd Dzul Hakim Wirzal, Muhammad Roil Bilad, Nik Abdul Hadi Md Nordin, Zulfan Adi Putra, Nonni Soraya Sambudi, Abdull Rahim Mohd Yusoff

**Affiliations:** 1 Chemical Engineering Department, Universiti Teknologi PETRONAS, Seri Iskandar, Perak 32610, Malaysia; nursyakinah94@gmail.com (N.S.A.H.); mroil.bilad@utp.edu.my (M.R.B.); nahadi.sapiaa@utp.edu.my (N.A.H.M.N.); zulfan.adiputra@utp.edu.my (Z.A.P.); soraya.sambudi@utp.edu.my (N.S.S.); 2 Faculty of Science, Universiti Teknologi Malaysia, Skudai 81310, Malaysia; arahimy@utm.my

**Keywords:** nanofiber membrane, solvent vapor, produced water

## Abstract

Electrospun nanofiber membrane (NFM) has a high potential to be applied as a filter for produced water treatment due to its highly porous structure and great permeability. However, it faces fouling issues and has low mechanical properties, which reduces the performance and lifespan of the membrane. NFM has a low integrity and the fine mat easily detaches from the sheet. In this study, nylon 6,6 was selected as the polymer since it offers great hydrophilicity. In order to increase mechanical strength and separation performance of NFM, solvent vapor treatment was implemented where the vapor induces the fusion of fibers. The fabricated nylon 6,6 NFMs were treated with different exposure times of formic acid vapor. Results show that solvent vapor treatment helps to induce the fusion of overlapping fibers. The optimum exposure time for solvent vapor is 5 h to offer full retention of dispersed oil (100% of oil rejection), has 62% higher in tensile strength (1950 MPa) compared to untreated nylon 6,6 NFM (738 MPa), and has the final permeability closest to the untreated nylon 6,6 NFM (733 L/m^2^.h.bar). It also took more time to get fouled (220 min) compared to untreated NFM (160 min).

## 1. Introduction

Rapid development of oil and gas industries lead to massive production of oily wastewater that can contaminate the environment without proper treatment. Generally, there are several conventional separation methods that are applicable for produced water (PW) treatment. Some of the methods are coagulation/flocculation [1,2], adsorption [3,4], hydrocyclone [5,6], and floatation [3,7]. All of them have merits and shortcomings and are more favourable than others under certain circumstances.

Membrane technology has emerged as one of the reliable and efficient remedies for treatment of oily wastewater [8]. It ensures stable quality of effluent and produces a small footprint. Nanofiber membrane (NFM) is one type of membrane that can be used to treat oily wastewater. NFM can be fabricated via melt blowing [9,10], phase separation [11,12], self-assembly [13,14], and electrospinning [15,16].

Electrospinning is one of the promising and reliable methods to produce nanofibers with diameter ranges of nanometers to micrometers. Electrospun nanofiber mats have huge surface area to volume ratio, high porosity, great water permeability and flexibility [17,18]. These attributes enable electrospun nanofibers to demonstrate their functionality and is widely applied in air filtration [19,20], water treatment [21,22], desalination [23,24], and adsorption [25,26].

However, the main drawbacks of using electrospun NFM are its fouling issues and its low mechanical strength [27]. Fouling causes pore blockage which increases filtration resistance and degrades hydraulic performance. Internal fouling can sometimes happen due to the penetration and trapping of small foulants between the overlapping layers of fibers. Moreover, the weak mechanical strength of NFM is due to its high porosity and weak bonding at the junctions of the fibers [28]. Apart from that, it is challenging to apply the NFM for filtration because of the defect-prone nature of the nanofiber mat layers, i.e., scratching. Under high pressure backwash, nanofiber mats can be easily delaminated and cannot be used anymore.

In this study, nylon 6,6 was chosen to be fabricated as NFM due to its high hydrophilicity, great tensile strength, and high thermal stability. However, as mentioned before, ENM has low mechanical strength which requires post treatment.

The fouling issue and weak mechanical property of NFM can be mitigated by incorporating nanofillers into the fiber or by implementing post treatment. A number of attempts have been carried out in order to improve the nanofibers bonding at the junctions of the fiber through post treatment, either chemically or physically. There are several techniques of post treatment which are heat-pressed treatments, coating treatments, and the cross-linking of fibers. Post treatments have been proven to alter the nanofiber properties as well as, to some extent, affecting material performance. For example, Yao et al. [29] reported on the implementation of annealing of NFM onto heat-pressed membranes for membrane distillation application. The performance increases by 10% with an average flux of 35 L/m^2^.h compared to the one without annealing, while 99.99% salt rejection can still be maintained. Cai et al. [30] crosslinked cellulose acetate nanofiber mats via solvent annealing where it is exposed to an ethanol/acetone mixture. After eight hours of exposure, the tensile strength and modulus improved by 102% and 33%, respectively. Moreover, Huang et al. [31] improved the mechanical properties and increased the hydrophilicity of electrospun polyaclonitrile and polysulfine NFMs by coating them with polydopamine for liquid based filtration. Coating improves hydrophilicity and increases mechanical strength up to three-fold for both NFMs, while still maintaining high water permeability.

Solvent vapor exposure has recently been prepared for post treatment of NFM, and its preferable thanks to more organized nanoscale phase separation and greater thermodynamic stable morphology of the active layer [32]. Apart from that, solvent vapor does not greatly change the morphology and dimension of the membrane and is less aggressive to weld fibers since residue solvent can help to ease the fusion between the fibers [28]. Furthermore, as solvent vapor treatment is applied, it allows the fibrous structure to be more compact where higher tensile strength could be achieved by increasing the density of nanofiber, fiber diameter, and crystallinity of polymers within fibers [15,30]. Hence, solvent vapor treatment of NFM can be seen as a necessary modification, as outlined by Huang et al. [29] that improves polysulfone NFM tensile strength from 0.8 MPa to 3.5 MPa. However, most of the previous studies only highlight the use of solvent vapor treatment in increasing NFM tensile strength, and not in terms of its performance for treating heavy duty waste. Despite reports on the application of solvent vapor to enhance mechanical strength on nanofiber mats, to our best knowledge, there are a lack of reports on its application on nylon 6,6. Most available reports also only focus on the effect of the treatment on morphological changes without comprehensive reports on filterability performance for treatment of real wastewater feed, as done in this study.

In this study, we apply solvent vapor exposure for post treatment of nylon 6,6 nanofiber mats for real PW filtration. It is expected that solvent vapor treatment promotes fusion and melting of fibers, which then produces membranes with a smoother surface which is then able to control the fouling rate and improves NFM tensile strength. In order to do that, firstly, we study the effect of exposure time of formic acid vaporization on the membrane properties. Next, we perform permeability and rejection analysis for each untreated and treated membrane. Later, we evaluate the results to identify the optimal exposure time for electrospun nylon 6,6 NFM.

## 2. Materials and Methods

### 2.1. Preparation of Nylon 6,6 Solution

The materials used in this study were formic acid, (98–100%, MERCK, Kenilworth, NJ, USA), glacial acetic acid, (99.85%, VWR Chemicals, Radnor, PA, USA), and nylon 6,6 pellets (Sigma Aldrich, St. Louis, MO, USA). A mixture of formic acid and acetic acid with a ratio of 1:1 was used to dissolve nylon 6,6 pellets (14.0 wt %) in order to prepare nylon 6,6 solution.

### 2.2. Electrospinning of Nylon 6,6 NFM

A 5 mL syringe was filled with nylon 6,6 solution and it was attached with a capillary tip of a 0.6 mm inner diameter. The feeding rate was set at a constant of 0.4 mL h^−1^. The voltage used was 26.0 kV and the distance from needle tip to a metal screen collector was 15 cm. Aluminum foil was placed on the rotator and the collector rotation was set at 500 RPM.

### 2.3. Solvent Vapor Treatment

After fabrication of the NFMs, they were cut into smaller pieces (2 cm × 2 cm). Thirty milliliters of formic acid was poured into a beaker and was then sealed inside a vacuum packed chamber together with the NFM pieces. The chamber was set to a vacuum of 70 kPa and the pressure was later built-up from formic acid evaporation. They were exposed to formic acid vapor for different periods of time (5, 12, 24 and 48 h) under room temperature, before collected and proceeded with characterization. The membrane samples were later labelled as S0, S5, S12, S24 and S48, depending on the exposure time. By applying solvent vapor treatment, it can induce the fusion of the fibers in order to form a physical bond at the contacting points of the nanofibers [33].

### 2.4. Membrane Characterization

The membrane was characterized in terms of surface morphology, porosity, hydrophilicity (contact angle), surface roughness and mechanical strength. A field emission scanning electron microscope (FESEM, Model: VPFESEM, Zeiss Supra55 VP, Feldbach, Switzerland) was used to observe the morphology of the membrane. All samples were mounted onto a metal substrate using carbon tape and coated with a thin layer of gold. For pore size measurement, ImageJ software was used. Furthermore, for porosity measurement, the dry wet method was used where the membrane weight and volume were measured. Membrane hydrophilicity (contact angle) was measured by using a goniometer via the Sessile Drop Method (IFT, Model: OCA 20, Data Physics, Filderstadt, Germany). To analyse surface roughness, an atomic force microscope (AFM, Model: NanoNavi E-Sweep Anton Paar, GmbH, Graz Austria) was used. The mechanical strength of the membrane was tested according to the ASTM standard D638 with a crosshead speed of 10 mm/min by using Universal Testing Machine (UTM, Shimadzu, Nakagyo-ku, Kyoto, Japan). The membranes were cut with a dimension of 30 mm × 70 mm and were mounted with an aluminum plate at both ends for a better grip.

### 2.5. Permeability Analysis

Permeability of electrospun NFM for pure water and PW were measured by using a cross-flow MF/UF testing unit operated at a constant feed pressure of 0.1 bar. The permeate was measured at 10 min intervals until it reached a steady-state value. A NFM with an area of 9 cm^2^ was placed at the membrane holder while water was pumped from the feed tank. The feed liquid was pumped at a constant cross flow velocity of 0.44 cm/s.

### 2.6. Rejection Analysis

Some of the samples were kept before and after the filtration test in order to conduct rejection analysis. Rejection analysis was divided under several tests, which are rejection of oil, total organic carbon (TOC) and turbidity. They were measured using UV-VIS Spectrophotometer (Model: DR 5000 Spectrophotometer, Hach Company, Loveland, CO, USA), Hach-Lange kits for COD, TOC analyzer (Model: TOC-VCSH, Shimadzu, Kyoto, Japan), and turbidity meter (Model: 2100Q Portable Turbidimeter, Hach Company, Loveland, CO, USA), respectively.

## 3. Results and Discussion

### 3.1. Membrane Properties

**Figure 1:**
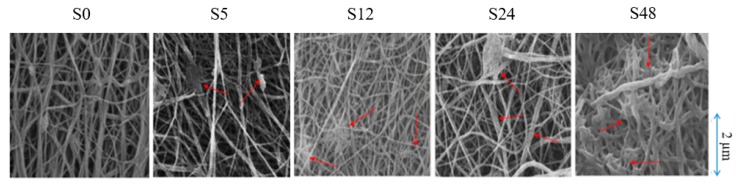
FESEM images for untreated nylon 6,6 NFM and treated nylon 6,6 NFM at treatment times of 5, 12, 24 and 48 h with magnification of 10,000×.

Figure 1 shows the field emission scanning electron microscope (FESEM) images for untreated and treated nylon 6,6 NFMs at treatment times of 5, 12, 24 and 48 h. The red arrows indicate the swelling phenomena which occur on the fibers. Swelling occurs as there is fusion between the fibers where the polymer of the membrane dissolved and melted with the help of solvent vapor [28,34]. The cross-linking of the fibers was also caused by the high volatility of formic acid. The fiber already started to swell after 5 h of exposure time and the occurrence of swelling increases as the exposure time increases. However, the membrane swelled completely when it was exposed for 48 h. As predicted, too much cross linking would occur when the membrane was exposed to formic acid vapor for a long period of time [33].

**Figure 2:**
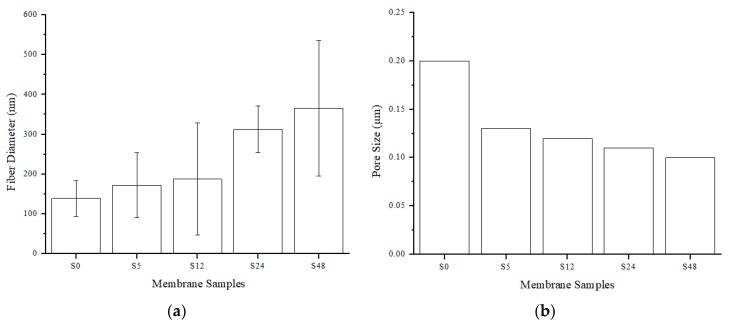
Fiber diameter (**a**) and pore size (**b**) as function of solvent vapor exposure time. The lines in the bars of represent the standard deviation.

Table 1 shows the membrane properties of nylon 6,6 NFMs. Solvent vapor treatment clearly affects the porosity, the pore size and the surface roughness (Table 1). Figure 2a,b represent the fiber diameter and pore size as a function of solvent vapor exposure time. The fusion and melting of fibers caused an incremental increase in fiber diameter, as can observed in Figure 2a, and reduction in membrane pore size (Figure 2b). This resulted in a porosity reduction whereby the porosity of nylon 6,6 NFM is reduced from 71.30 ± 2.00% to 62.00 ± 1.00% when it is exposed for 48 h. The pore size decreases from 0.2 µm for S0 to 0.1 µm for S48 NFM.

**Figure 3:**
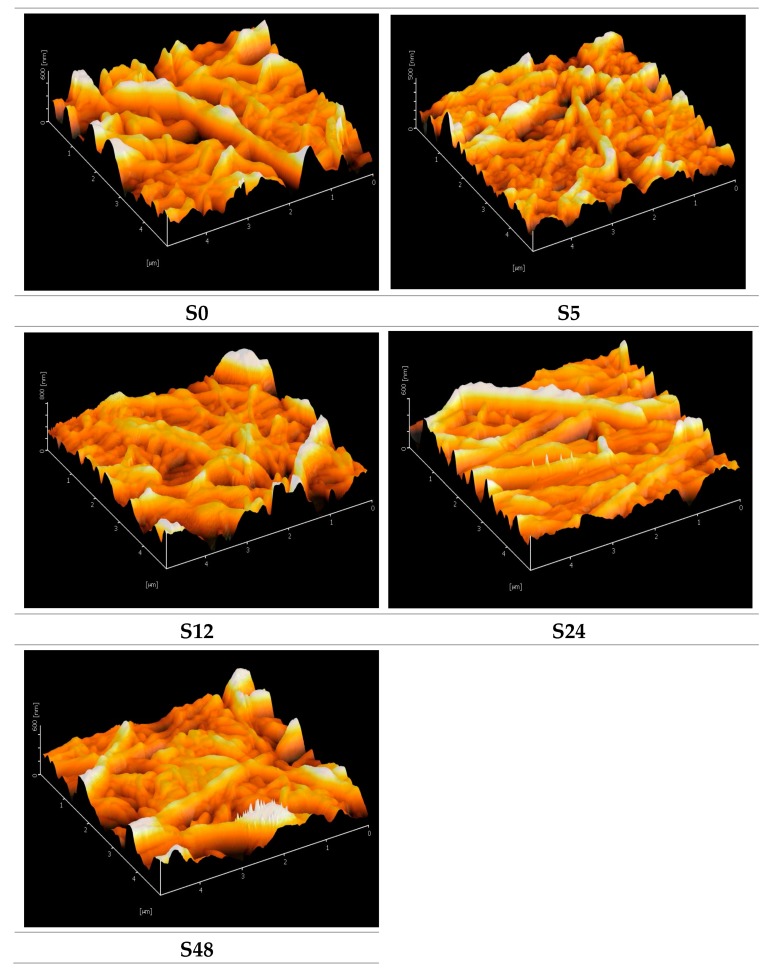
AFM images for untreated nylon 6,6 NFM and treated nylon 6,6 NFM at treatment times of 5, 12, 24 and 48 h.

Figure 3 shows AFM images for untreated nylon 6,6 NFM and treated nylon 6,6 NFMs. The solvent vapor treatment reduces the surface roughness of nylon 6,6 NFMs and the effect is more profound as the exposure time increases, as can be seen in Figure 3 and Table 1. This phenomenon occurs as surface tension overpowers surface viscosity due to swelling (vapor is absorbed to the membrane surface). The high peaks of surface roughness will “run” through the valleys driven by the surface tension and hence resulted in a smoother surface [35,36]. Surface roughness plays an important role in controlling the fouling in membrane separation. Membrane that has a rougher surface tends to get fouled easier due to an accumulation of deposits on the valleys [37].

**Figure 4:**
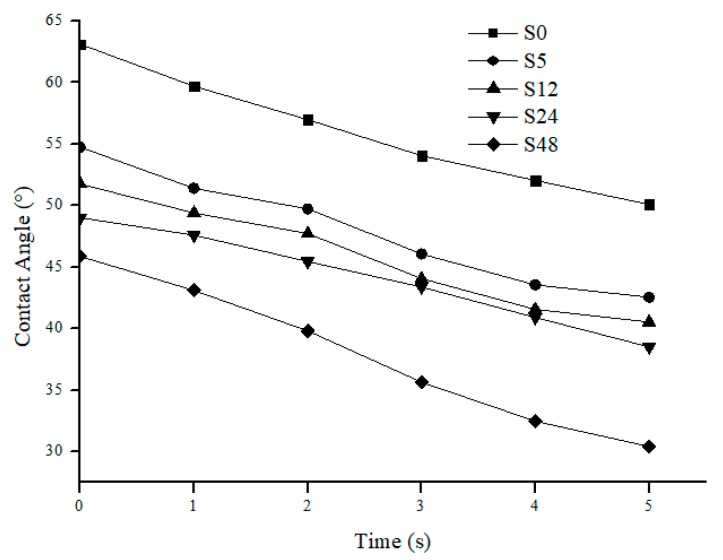
Dynamic contact angle vs. time for untreated nylon 6,6 NFM and treated nylon 6,6 NFM with treatment time at 5, 12, 24 and 48 h.

Figure 4 shows water contact angle vs. time for nylon 6,6 NFM treated with solvent vapor. The membrane hydrophilicity increases as the exposure time to solvent vapor increases. S48 membrane has the lowest dynamic contact angle. This reduction of contact angle is correlated with the increase in smoothness of the membrane surface as exposure time increases. Apart from that, the hydrophilic membrane is more preferable to reduce fouling since it is more resistant towards foulant which is usually hydrophobic in nature [38].

**Figure 5:**
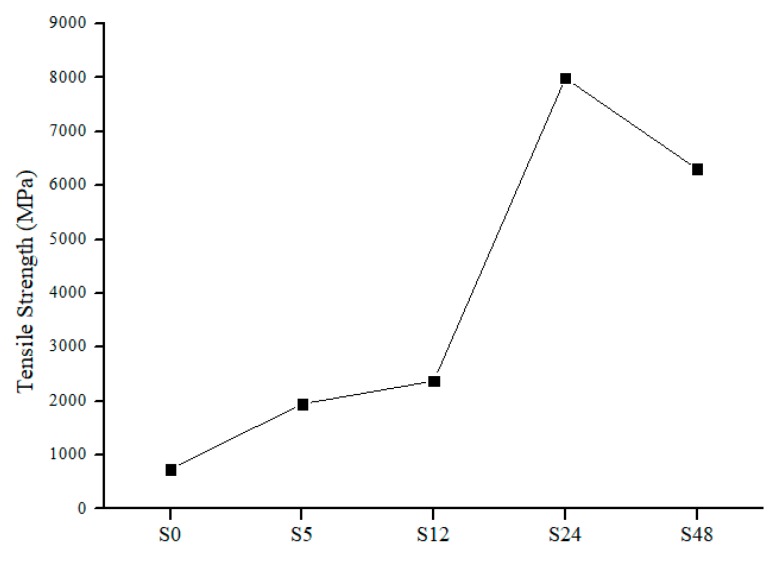
Tensile strength of nylon 6,6 NFMs.

The tensile strength of nylon 6,6 NFM after solvent vapor treatment is illustrated in Figure 5. The tensile strength for S0 is 737 MPa, respectively. After treatment of solvent vapor, the tensile strength increases gradually with an increase in exposure time. The tensile strength increases by 62%, 69%, and 91% for S5, S12 and S24, respectively. The increment in tensile strength is due to fusion at the junction of nanofibers [39,40]. The solvent vapor treatment promotes fusion between the interfiber junctions due to condensation of formic acid vapor. The longer the treatment time, the greater the number of junctions that can be induced by the vapor (the higher the number of crosslinking between fibers). The increase in tensile strength was also correlated with FESEM images (Figure 1). It also enlarges the fibers by merging them into different sites. Apart from that, solvent vapour treatment promotes random fusion across the NFM. By having random fusion, it will give a larger distribution of tensile strength, hence increasing NFM tensile strength.

Overexposure to solvent vapor causes damage to the NFM and thus will affect the mechanical strength of NFM [15] as shown for S48. The tensile strength decreases when the exposure time was set for 48 h. Overexposure to solvent vapour promotes fusion between fiber layers which is caused by uncontrolled swelling. This results in a small distribution of tensile strength among fibers. From a mechanical strength perspective, it can be concluded that the optimum exposure time for nylon 6,6 NFM would be 24 h, where it has the highest tensile strength and a 91% increment, as compared to S0.

### 3.2. Permeability Analysis

**Figure 6:**
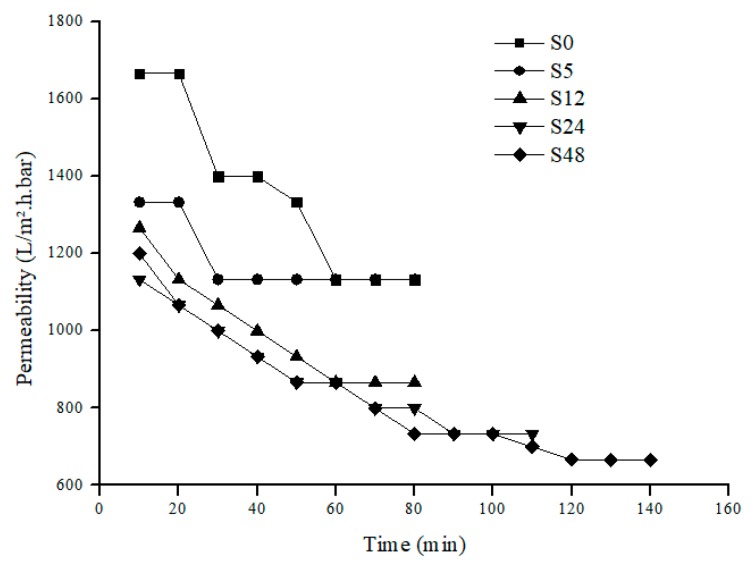
Pure water permeability of nylon 6,6 NFMs.

**Figure 7:**
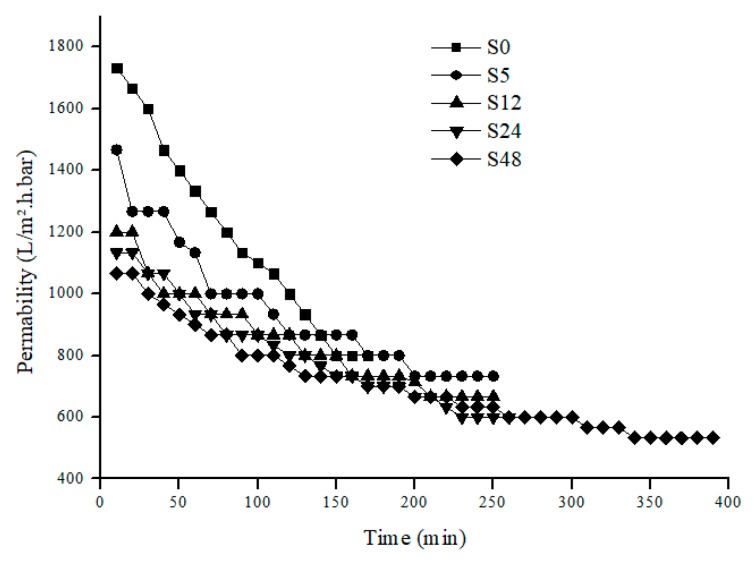
Produced Water permeability for nylon 6, 6 NFMs.

**Figure 8:**
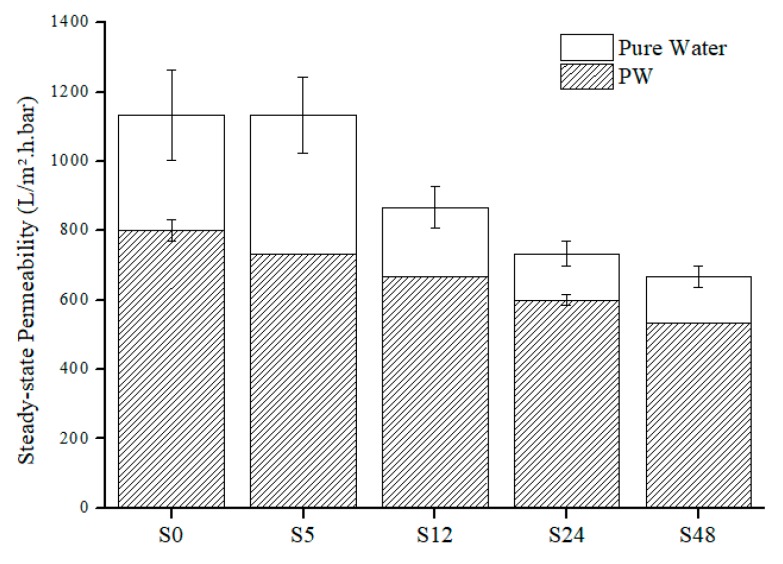
Steady-state pure water and PW permeability for nylon 6,6 NFMs.

For permeability analysis, it is based on overall and steady-state permeability of pure water and PW feed. Based on Figure 6 and Figure 7, both pure water and PW show the same trend of permeability where the permeability decreases as the treatment time increases. Like the overall permeability, both steady-state permeability of pure water and PW share the same trend (Figure 8). For steady-state permeability of PW, the highest was achieved by untreated nylon 6,6 NFM (800 L/m^2^.h.bar). Meanwhile, S48 has the lowest steady-state permeability of PW (533 L/m^2^.h.bar). This correlates with the FESEM images (Figure 1) and also the membrane properties (Table 1) where the membrane pore size becomes smaller as the treatment time increases due to the melting and fusion of fiber which then reduces the porosity and permeability of the membrane.

Nevertheless, interestingly, all treated NFM took a longer time to reach steady-state compared to an untreated one showing lower membrane fouling propensity. This can be clearly observed in Figure 7 where S48 took the longest time (340 min) to achieve steady-state or, in other words, took the longest time to get fouled. This indicates that solvent vapor treatment can help to delay the accumulation of foulants on the membrane surface since it increases hydrophilicity and also reduces membrane surface roughness. Despite the reduction of permeability when solvent vapor treatment is applied, by reducing fouling, it will prolong the membrane life and also reduce the membrane operational costs [41].

### 3.3. Rejection Analysis

NFM treated with solvent vapor able to achieve 100% of oil rejection rate and excellent decrease in turbidity (Table 2). Nylon 6,6 NFM treated with solvent vapor is also able to meet the Malaysia standard B discharge requirement for total oil and grease since no concentration of oil was found. Due to a reduction in the size of pores, smaller size particles were able to be filtered by the NFMs. Apart from that, the higher rejection of oil for the treated NFMs can be related with the increase of membrane hydrophilicity, where it favors water to flow through it and hence is able to reject more oil molecules. However, same as untreated nylon 6,6 NFM, for TOC, it does not meet the Malaysia standard B discharge requirement, since it is higher than 250 ppm.

## 4. Conclusions

The effect of solvent vapor treatment on electrospun nylon 6,6 NFM for PW treatment was investigated. The solvent vapor treatment was able to promote the physical fusion of fibers to occur at the nanofiber junction. It eventually reduces membrane pore size, porosity and permeability. Despite those disadvantages, by reducing membrane pore size, it gives excellent rejection of oil. Based on the results, the optimum exposure time for solvent vapor would be 5 h as it is sufficiently adequate to achieve full retention of dispersed oil (100% of oil rejection), 62% higher in tensile strength (1950 MPa) compared to untreated nylon 6,6 NFM (738 MPa), and has the final permeability closest to nylon 6,6 NFM (733 L/m^2^.h.bar). S5 membrane also took a longer time to get fouled (220 min) compared to untreated NFM (160 min). With these features, the NFMs can still achieve high permeability with full rejection of oil.

## Figures and Tables

**Table 1:** Membrane properties of nylon 6,6 NFMs.

Treatment Time (hours)	Sample Name	Porosity (%)	Pore Size (µm)	Surface Roughness (nm)
**0**	S0	71.30 ± 2.00	0.20	231.10
**5**	S5	70.00 ± 1.00	0.13	90.84
**12**	S12	69.00 ± 0.50	0.12	85.43
**24**	S24	64.00 ± 0.50	0.11	80.63
**48**	S48	62.00 ± 1.00	0.10	74.60

**Table 2:** Rejection analysis for nylon 6,6 NFMs.

Sample Name	Turbidity (NTU)	TOC (ppm)	Oil Conc. (ppm)	Oil Rejection Rate (%)
**PW (feed)**	33.50	583.00	88.43	-
**S0**	1.88	572.60	4.93	94.40
**S5**	0.25	573.80	0.00	100.00
**S12**	0.23	553.50	0.00	100.00
**S24**	0.20	573.10	0.00	100.00
**S48**	0.17	579.70	0.00	100.00
